# Supplementary figures and images for: CREB5 reprograms FOXA1 nuclear interactions to promote resistance to androgen receptor-targeting therapies
Source: eLife. 2022 May 12;11:e73223. doi: 10.7554/eLife.73223 (PMC9135408; doi:10.7554/eLife.73223)

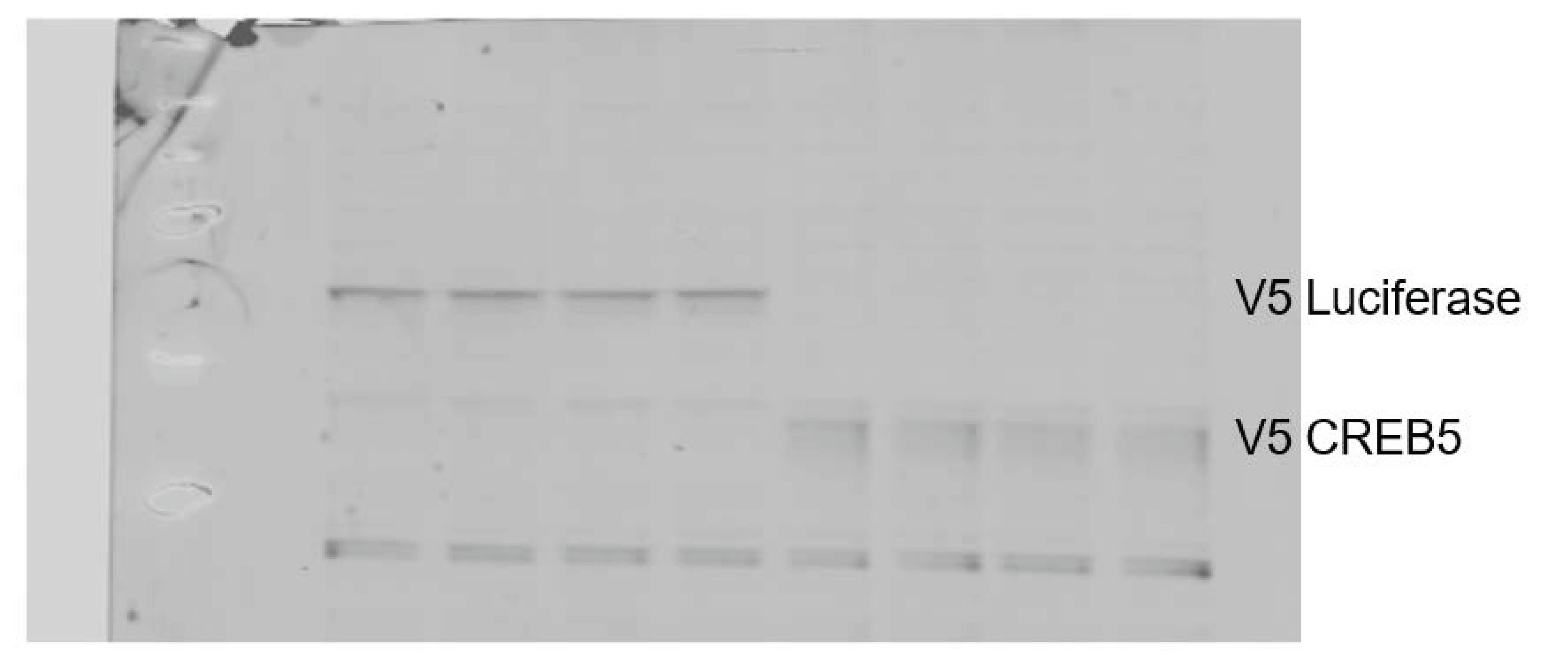

Supplement: Figure 1—figure supplement 1—source data 1. [file elife-73223-fig1-figsupp1-data1.tif]

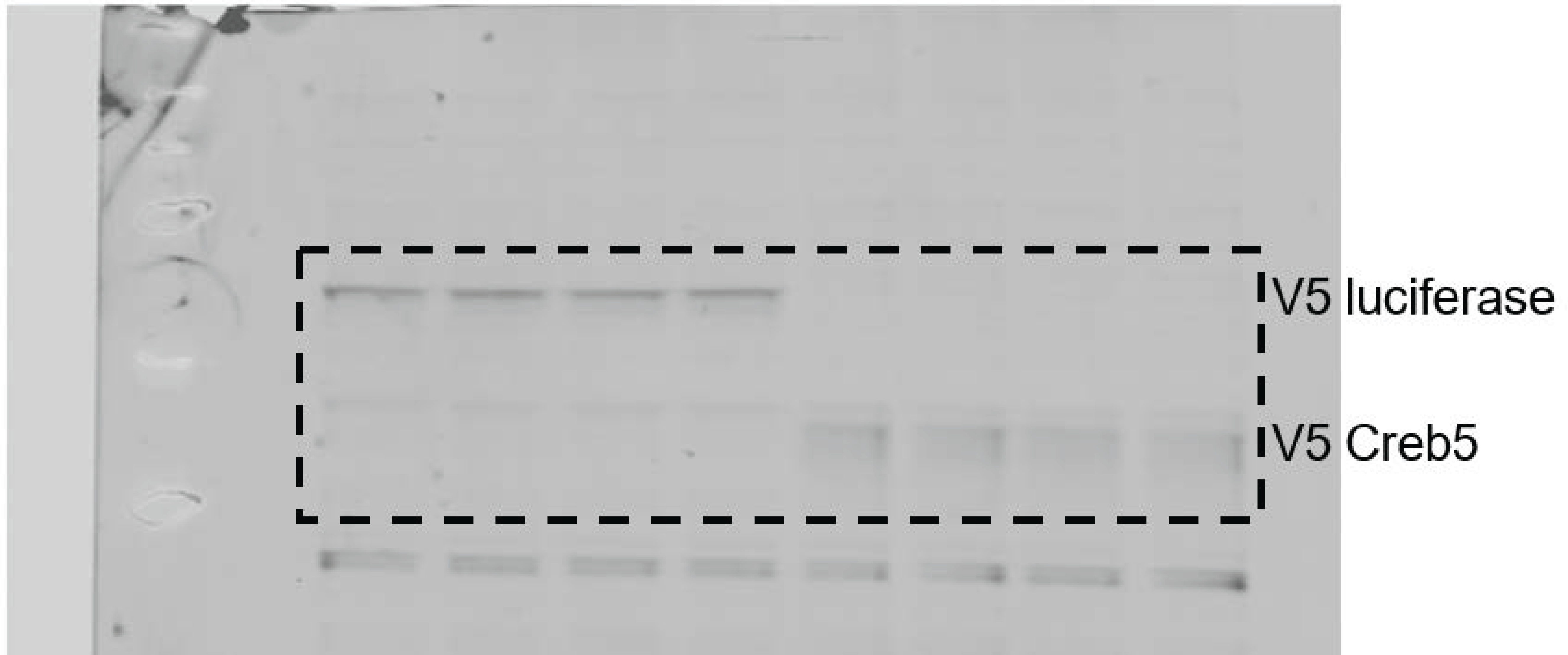

Supplement: Figure 1—figure supplement 1—source data 2. [file elife-73223-fig1-figsupp1-data2.tif]

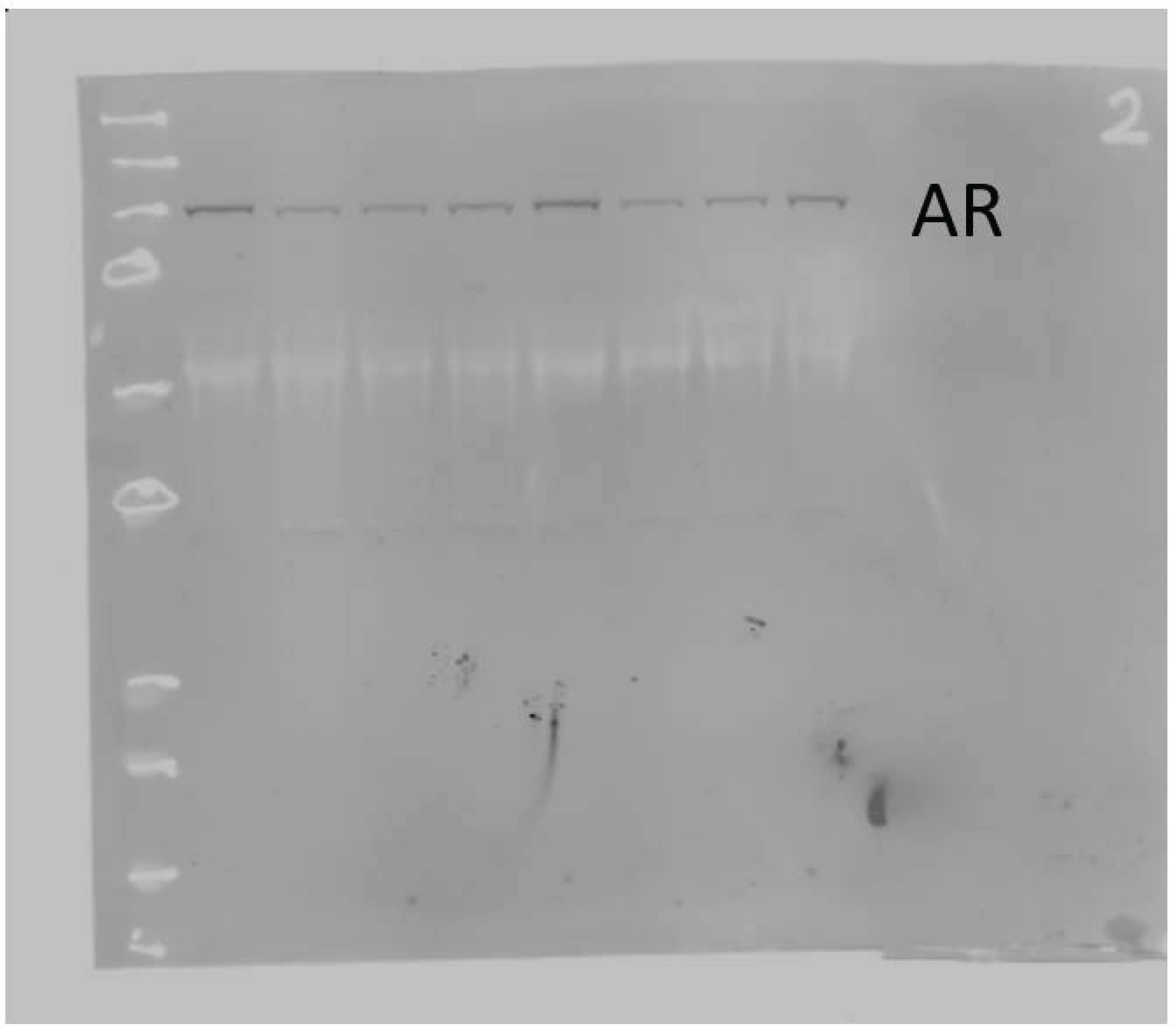

Supplement: Figure 1—figure supplement 1—source data 3. [file elife-73223-fig1-figsupp1-data3.tif]

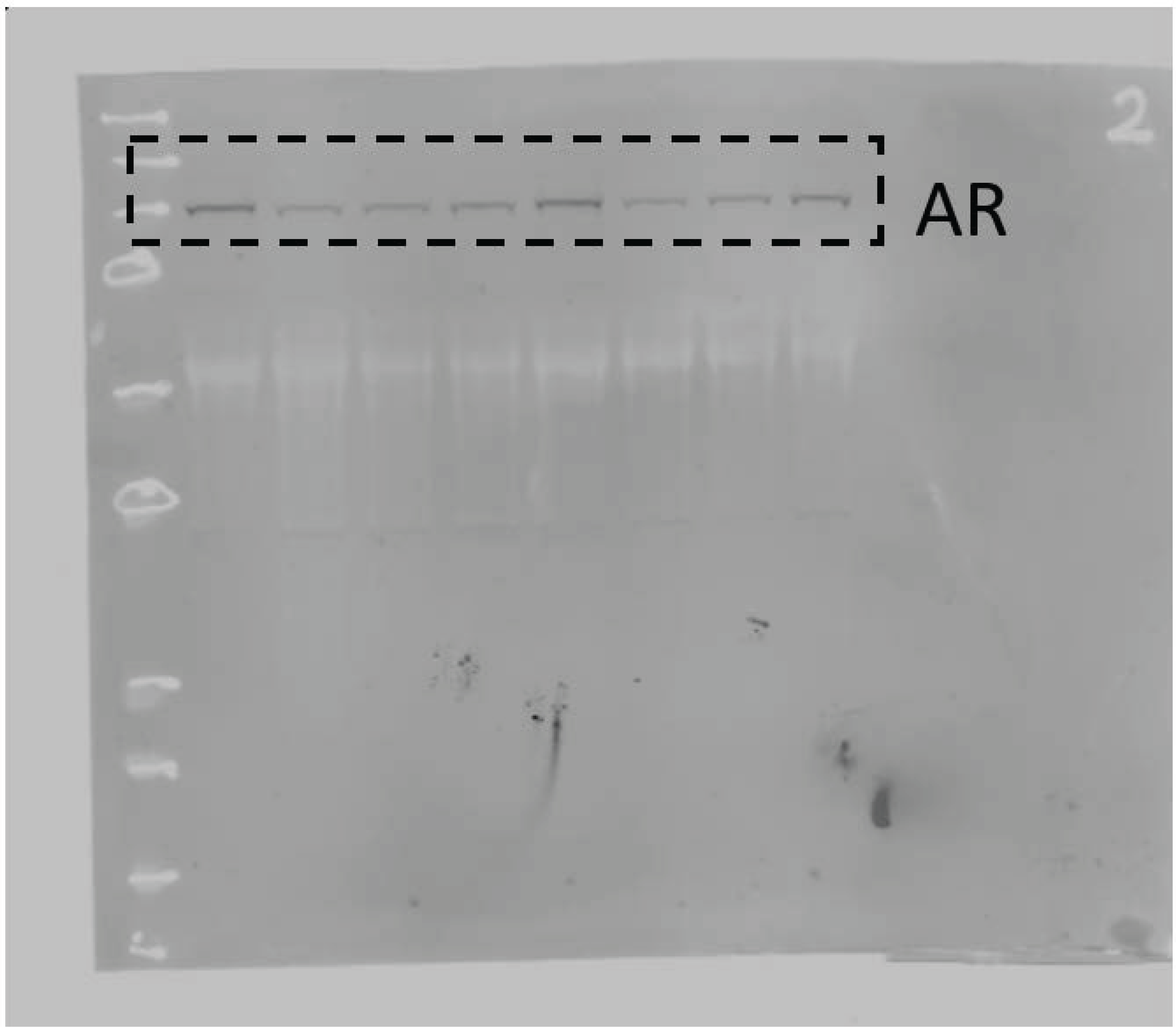

Supplement: Figure 1—figure supplement 1—source data 4. [file elife-73223-fig1-figsupp1-data4.tif]

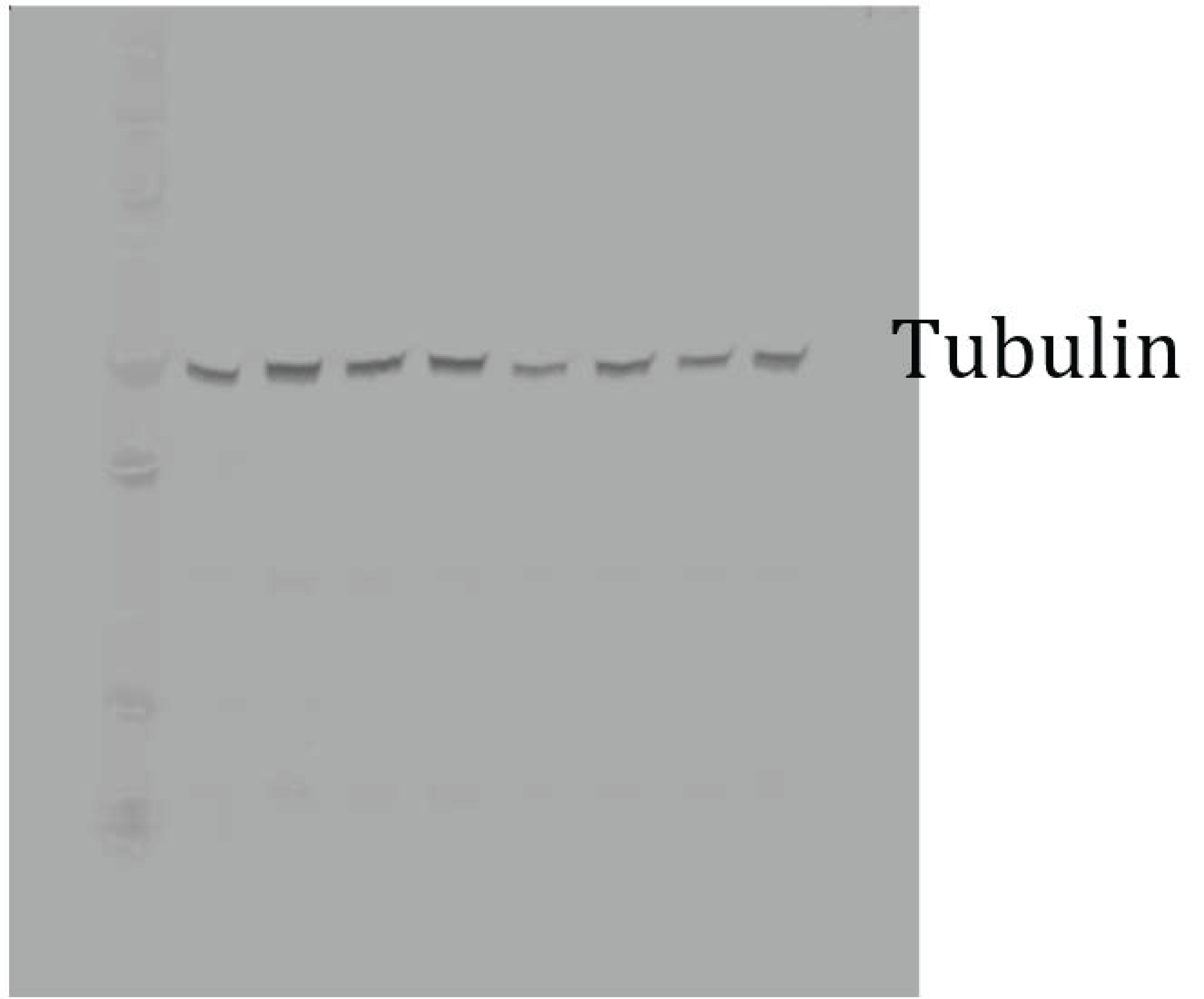

Supplement: Figure 1—figure supplement 1—source data 5. [file elife-73223-fig1-figsupp1-data5.tif]

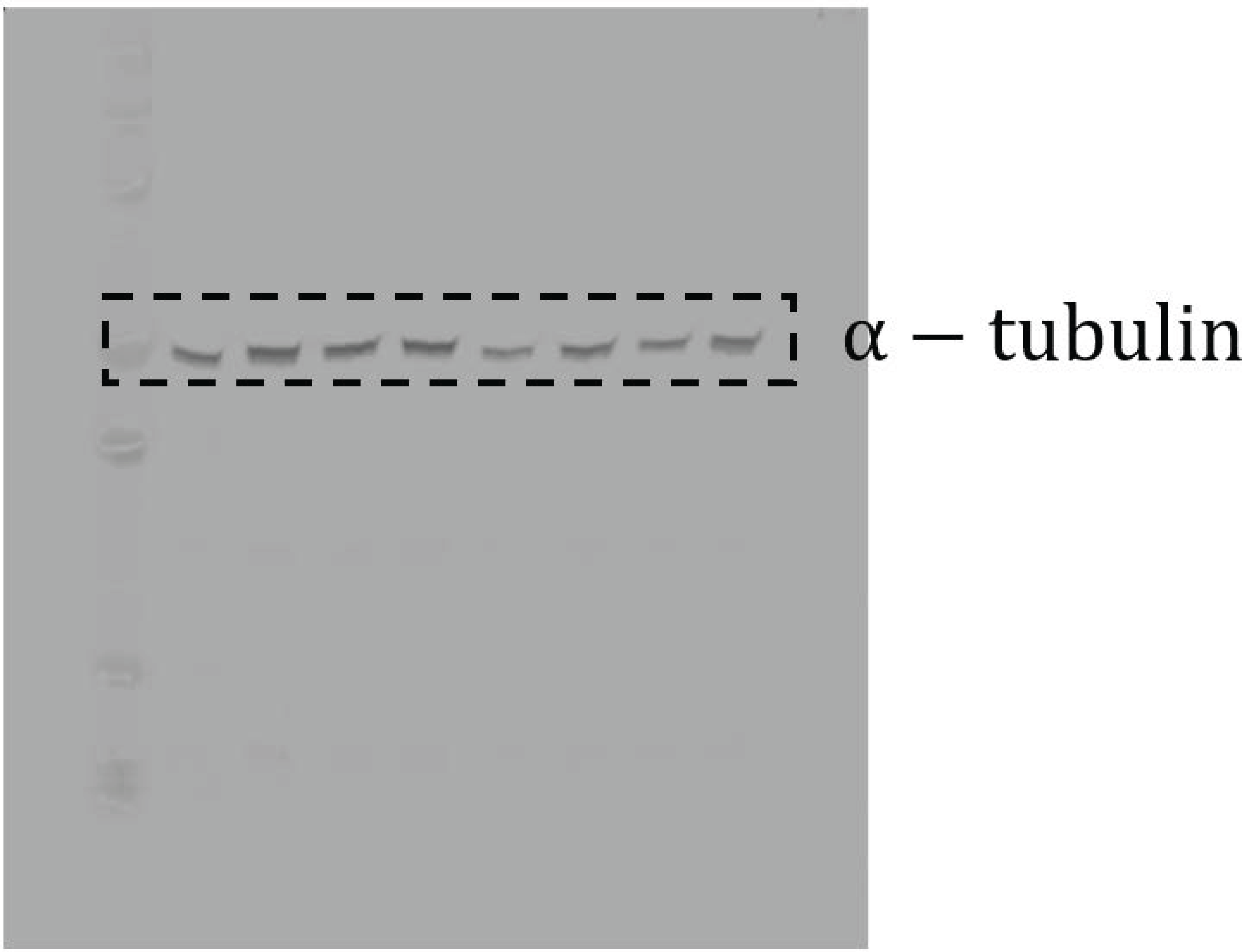

Supplement: Figure 1—figure supplement 1—source data 6. [file elife-73223-fig1-figsupp1-data6.tif]

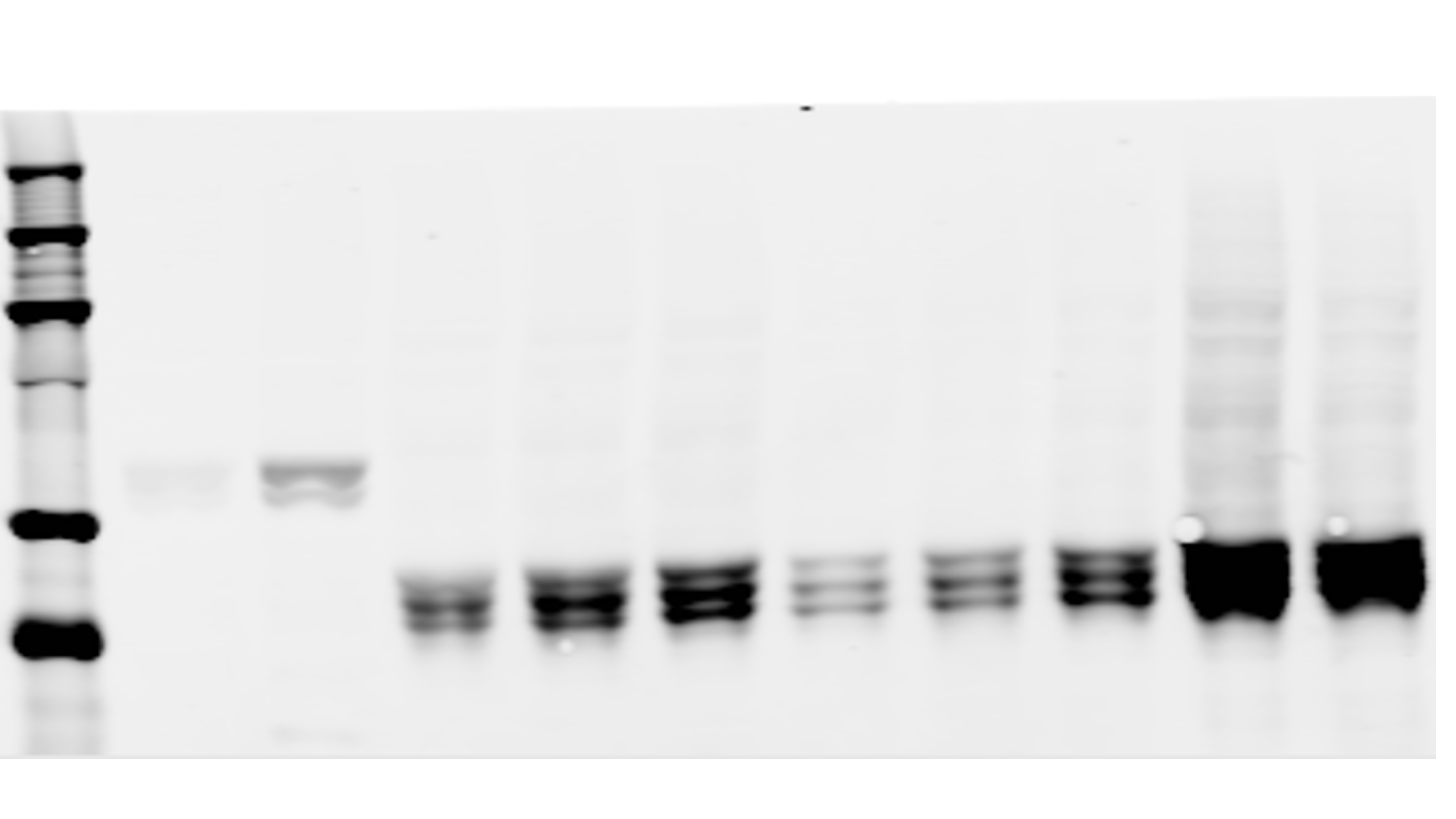

Supplement: Figure 3—source data 1. [file elife-73223-fig3-data1.tif]

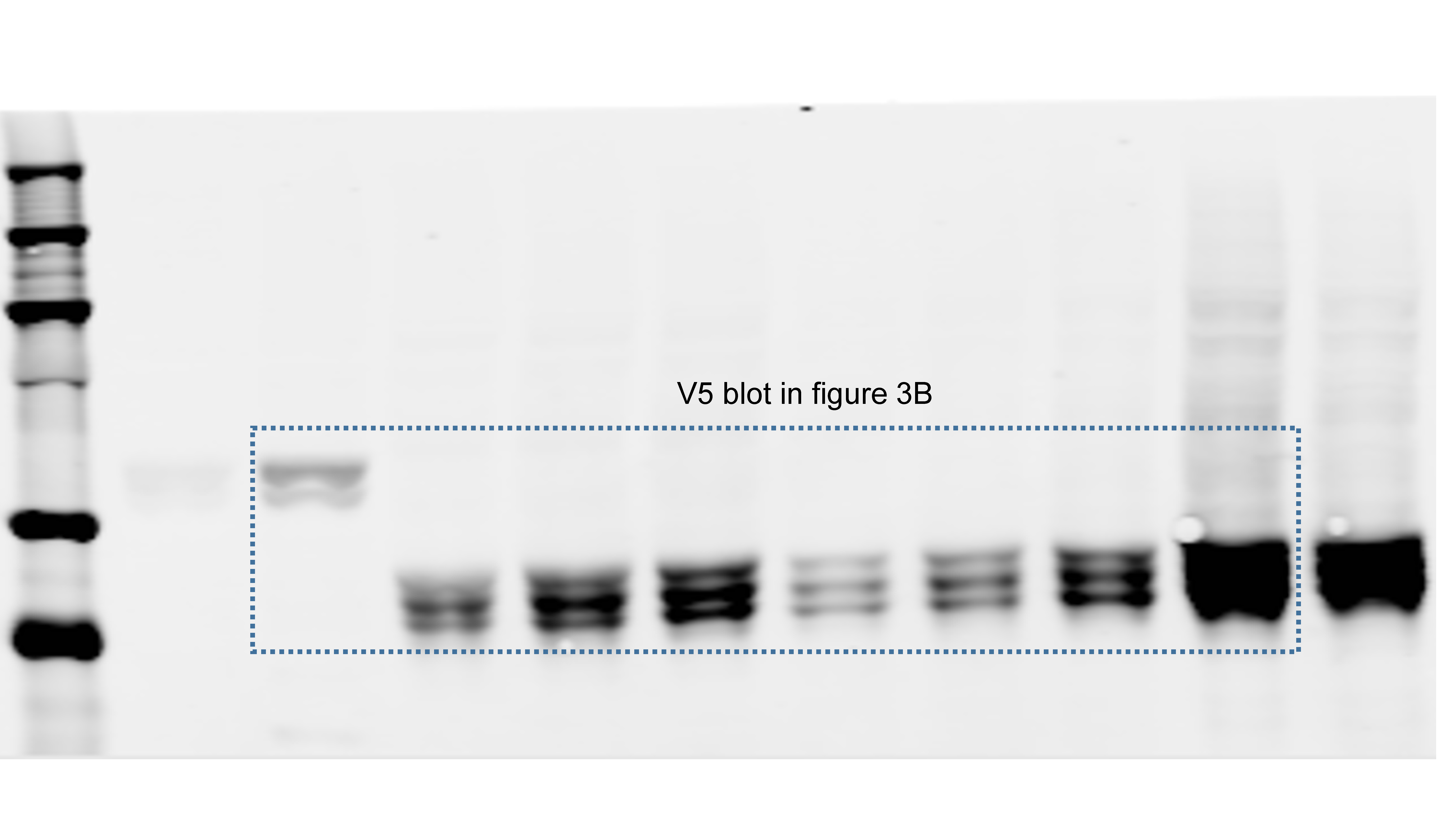

Supplement: Figure 3—source data 2. [file elife-73223-fig3-data2.tif]

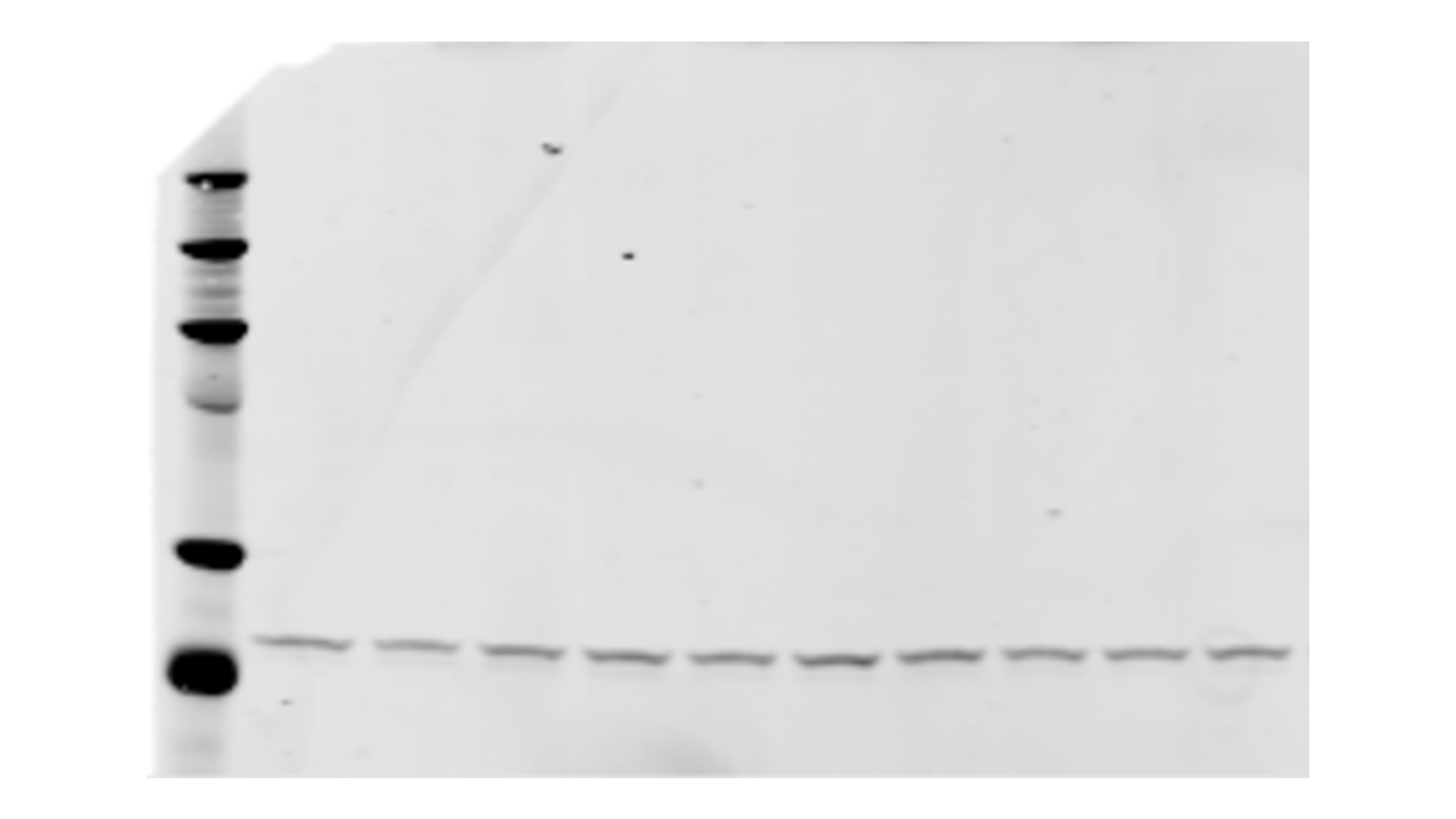

Supplement: Figure 3—source data 3. [file elife-73223-fig3-data3.tif]

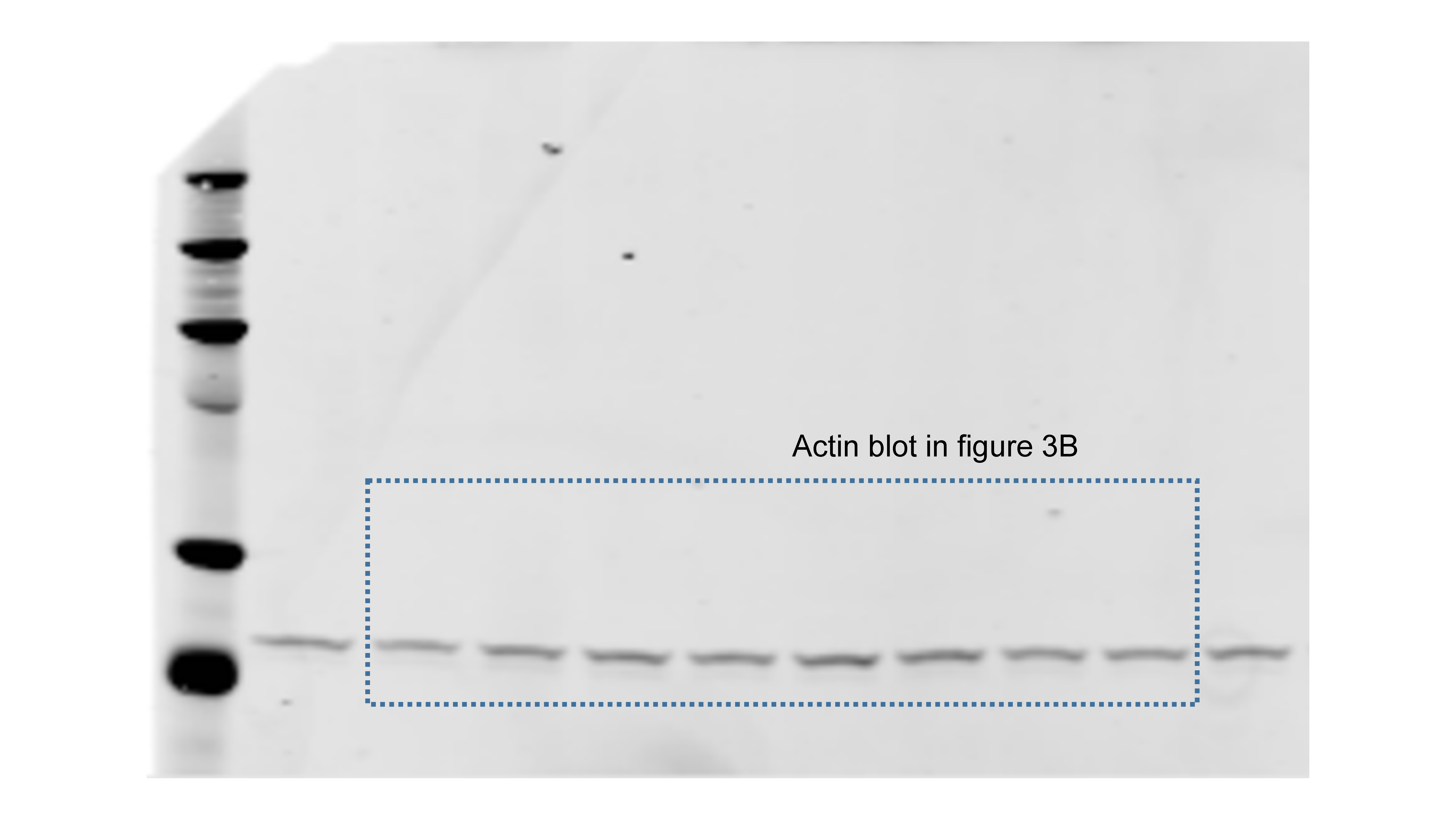

Supplement: Figure 3—source data 4. [file elife-73223-fig3-data4.tif]

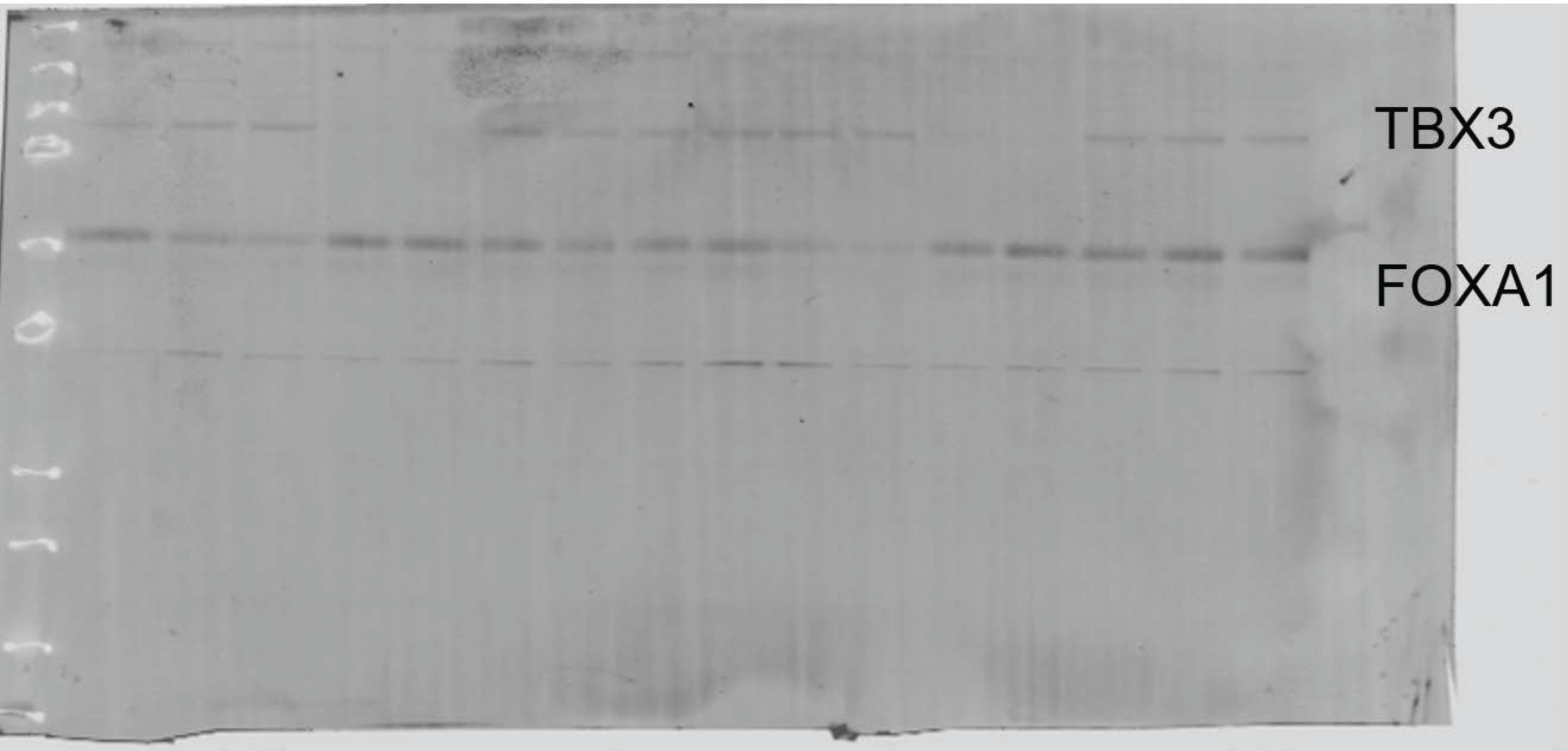

Supplement: Figure 4—source data 1. [file elife-73223-fig4-data1.zip › Figure 4 - Source Data 1.tif]

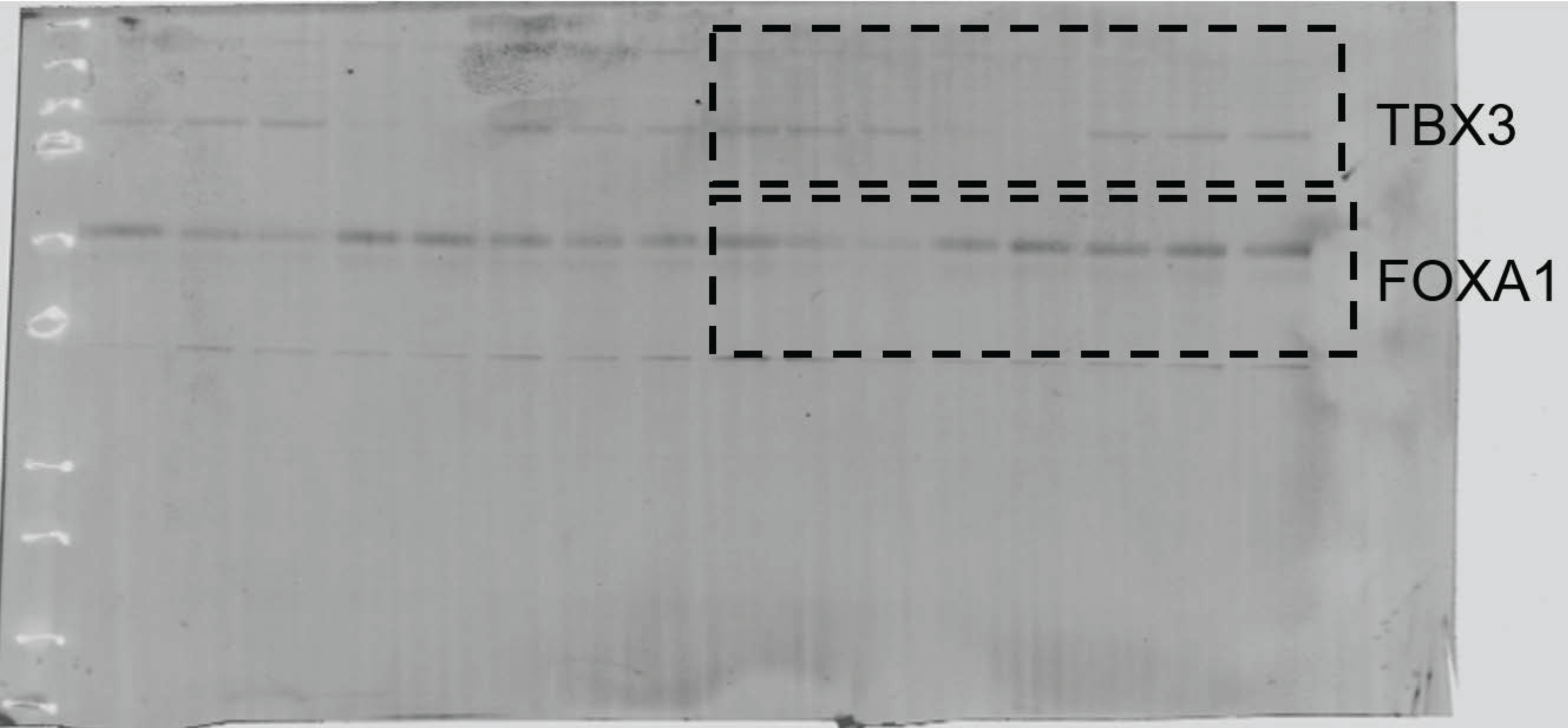

Supplement: Figure 4—source data 2. [file elife-73223-fig4-data2.zip › Figure 4 - Source Data 2.tif]

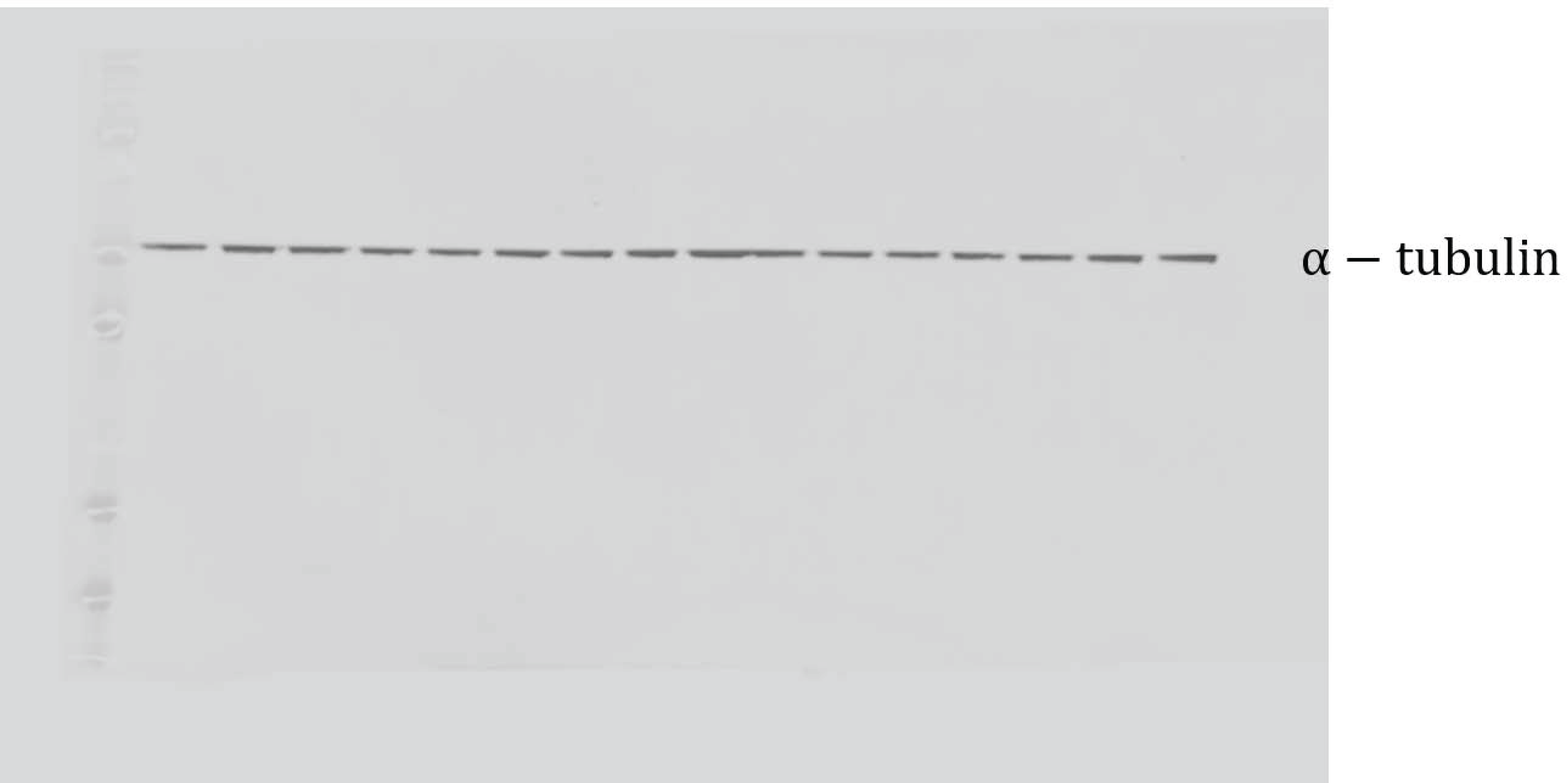

Supplement: Figure 4—source data 3. [file elife-73223-fig4-data3.zip › Figure 4 - Source Data 3.tif]

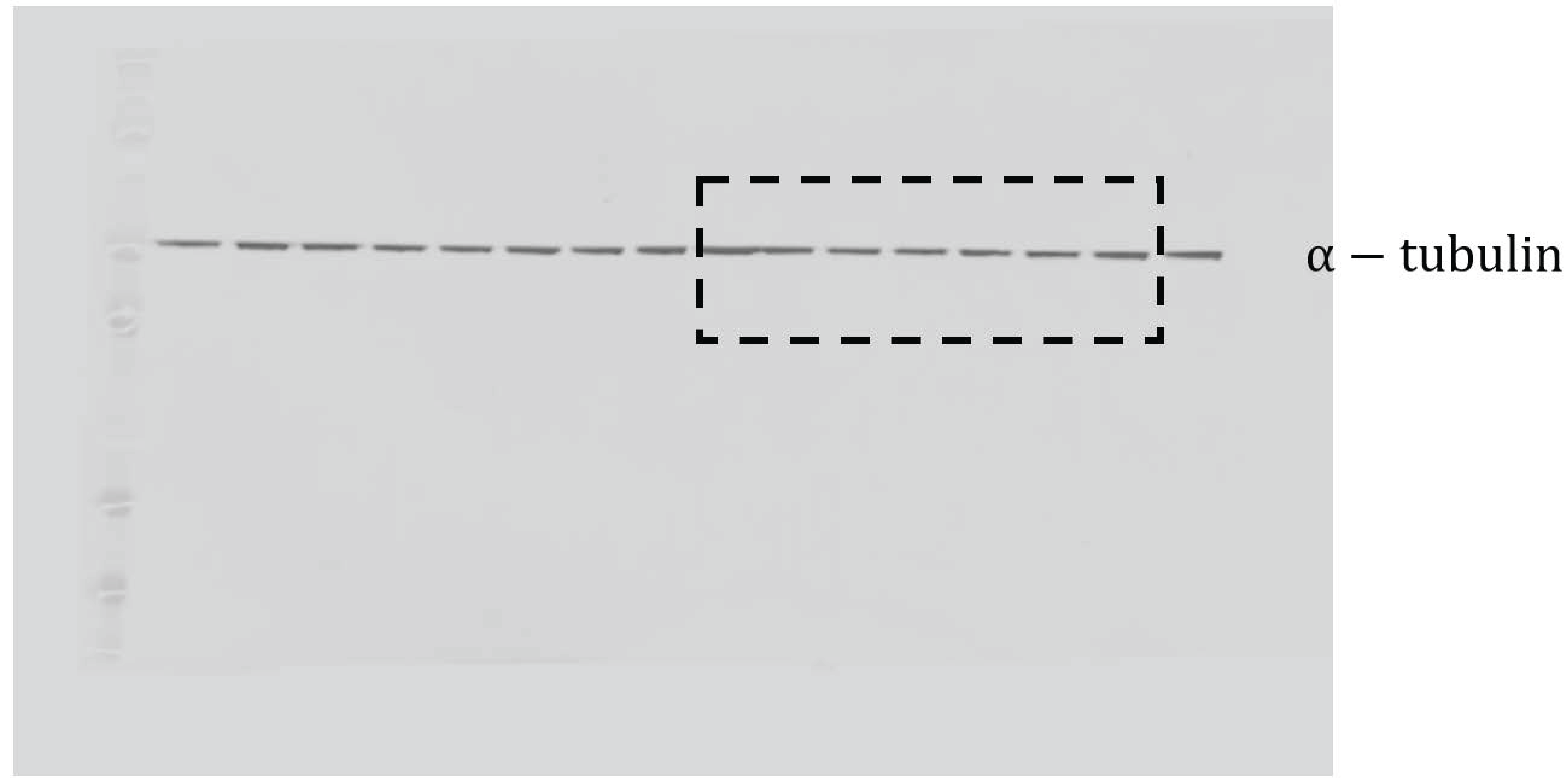

Supplement: Figure 4—source data 4. [file elife-73223-fig4-data4.zip › Figure 4 - Source Data 4.tif]

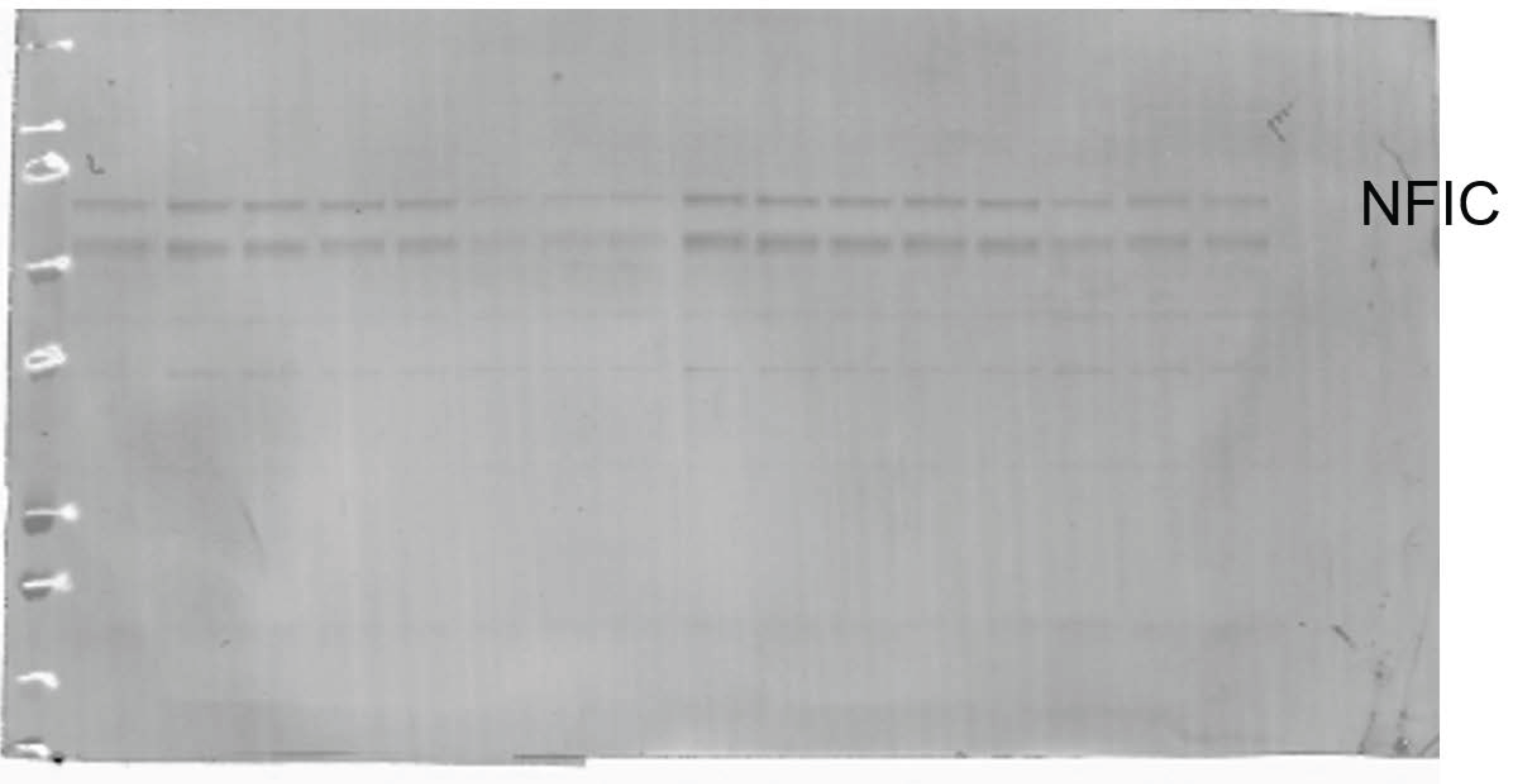

Supplement: Figure 4—source data 5. [file elife-73223-fig4-data5.tif]

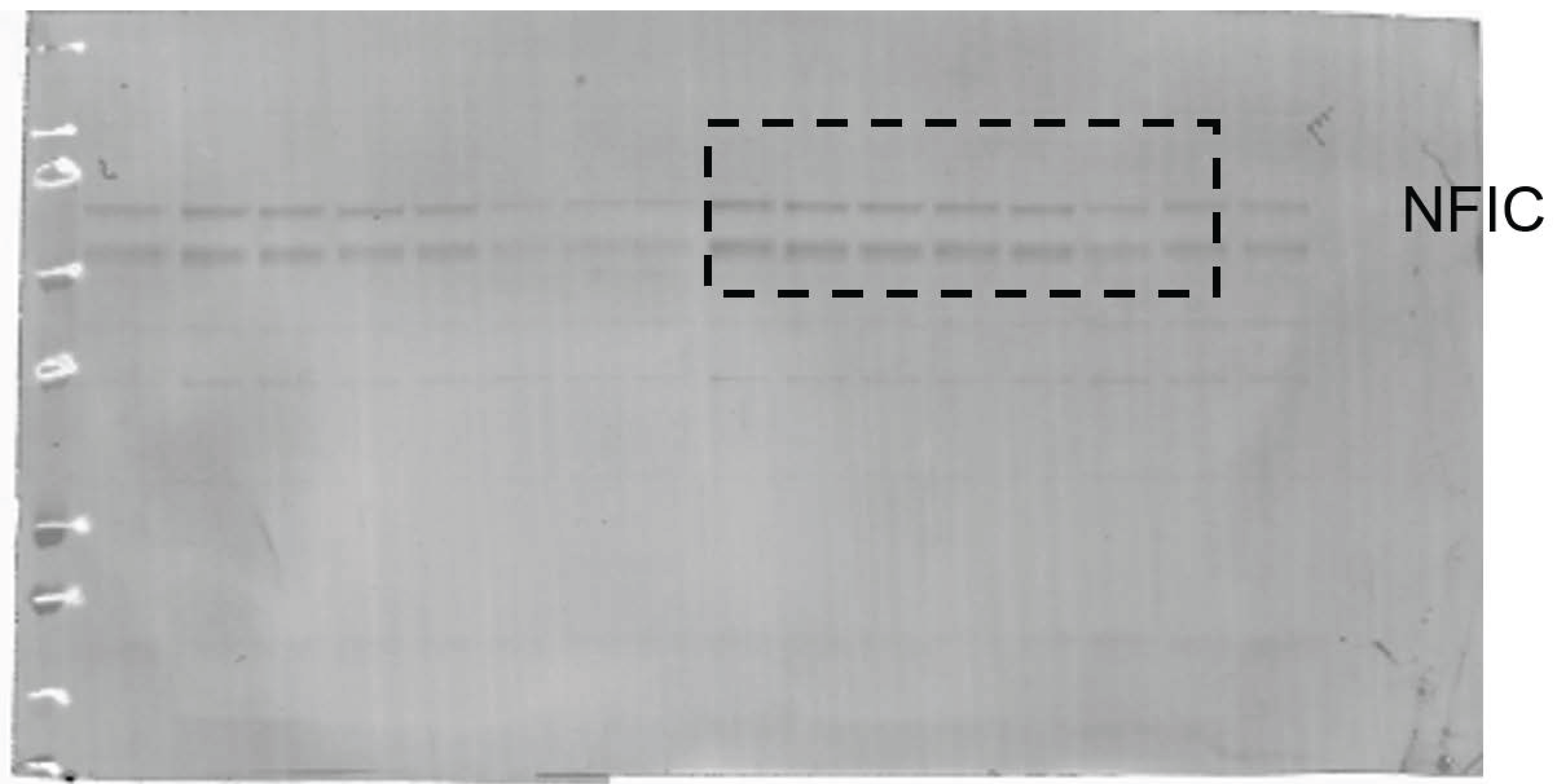

Supplement: Figure 4—source data 6. [file elife-73223-fig4-data6.tif]

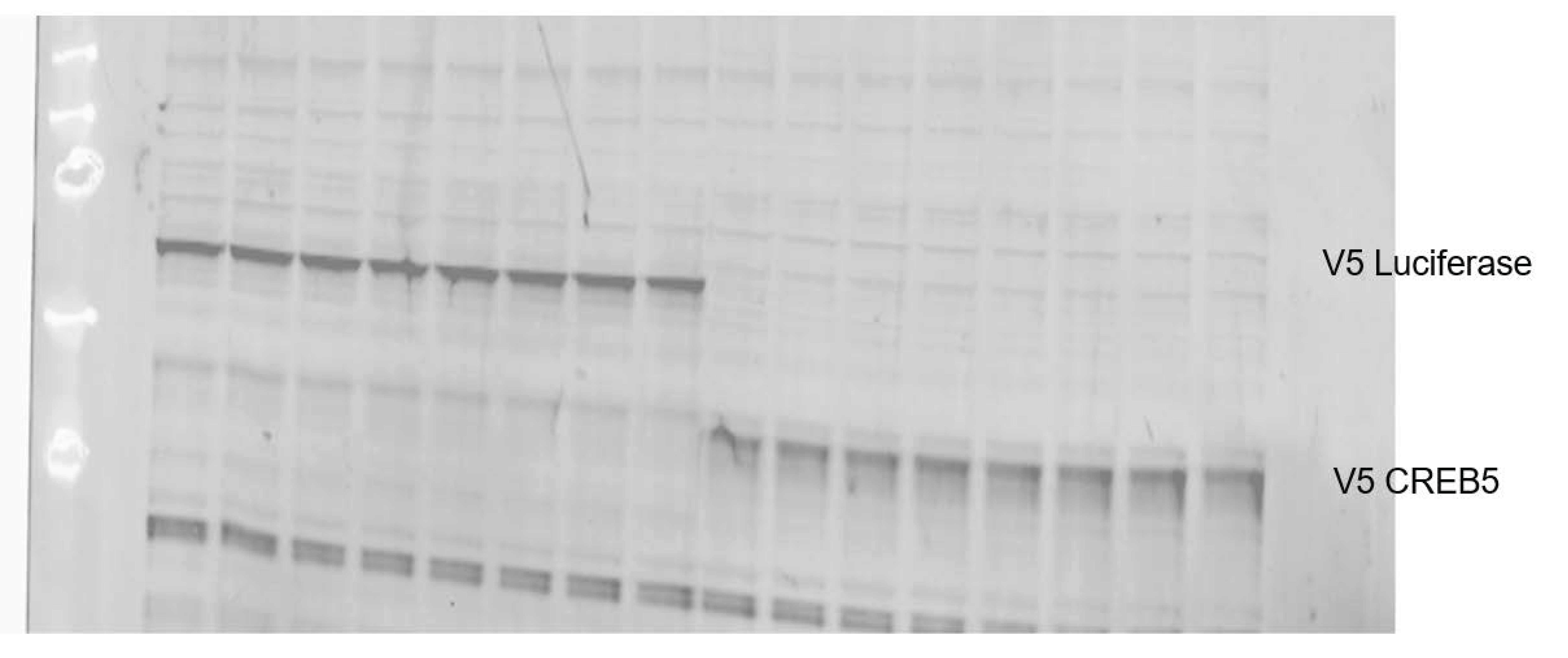

Supplement: Figure 4—source data 7. [file elife-73223-fig4-data7.tif]

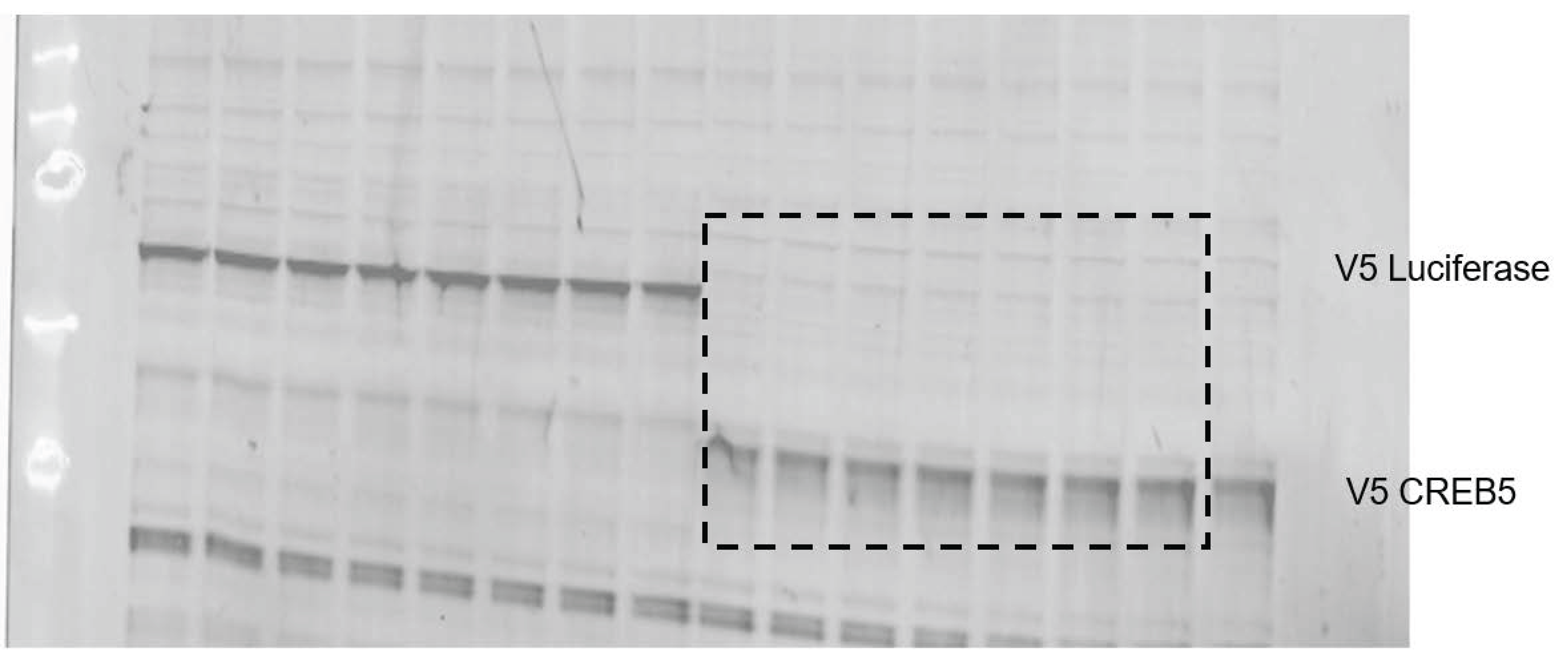

Supplement: Figure 4—source data 8. [file elife-73223-fig4-data8.tif]

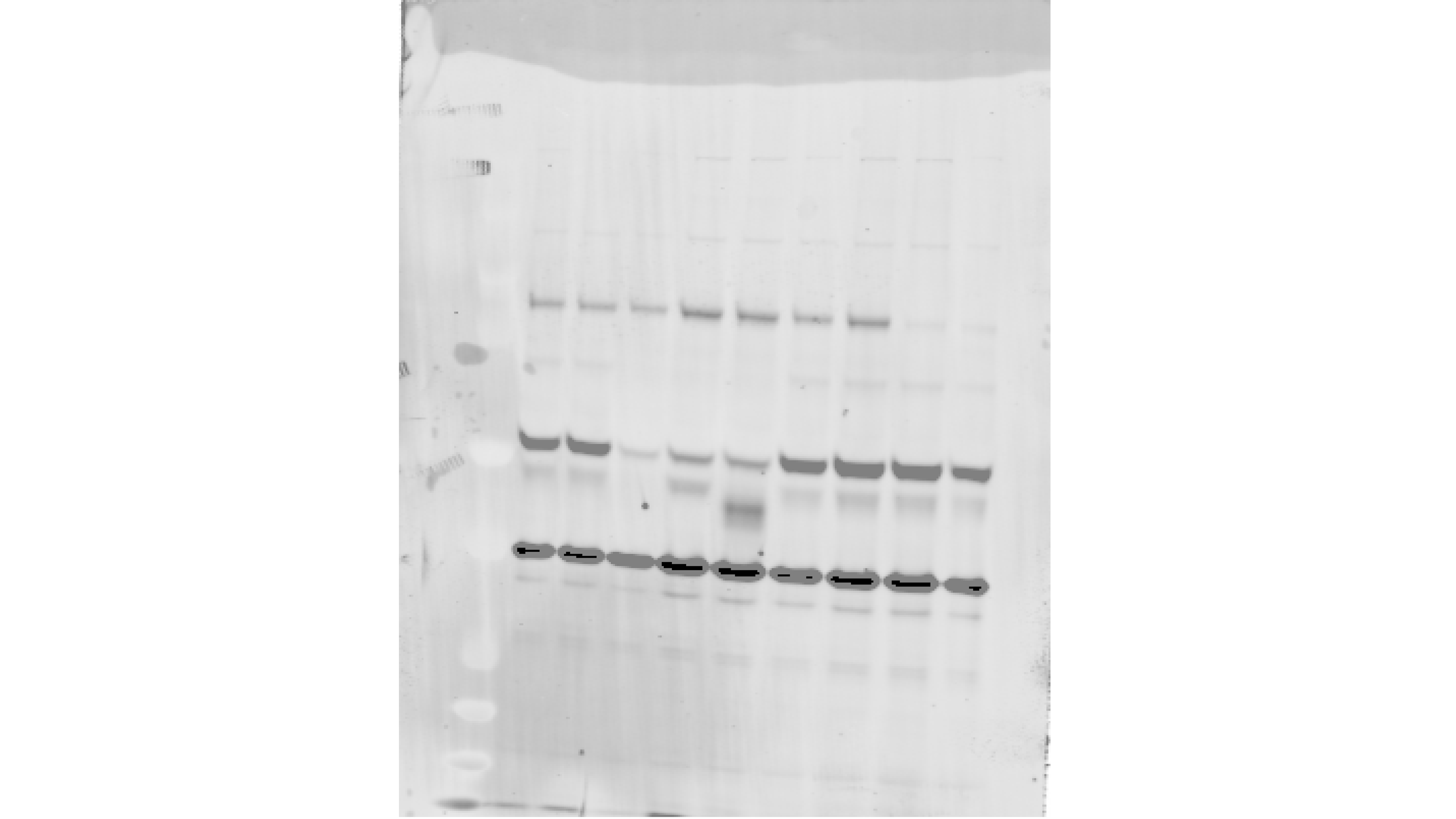

Supplement: Figure 4—source data 9. [file elife-73223-fig4-data9.zip › Figure 4 - Source Data 9.tif]

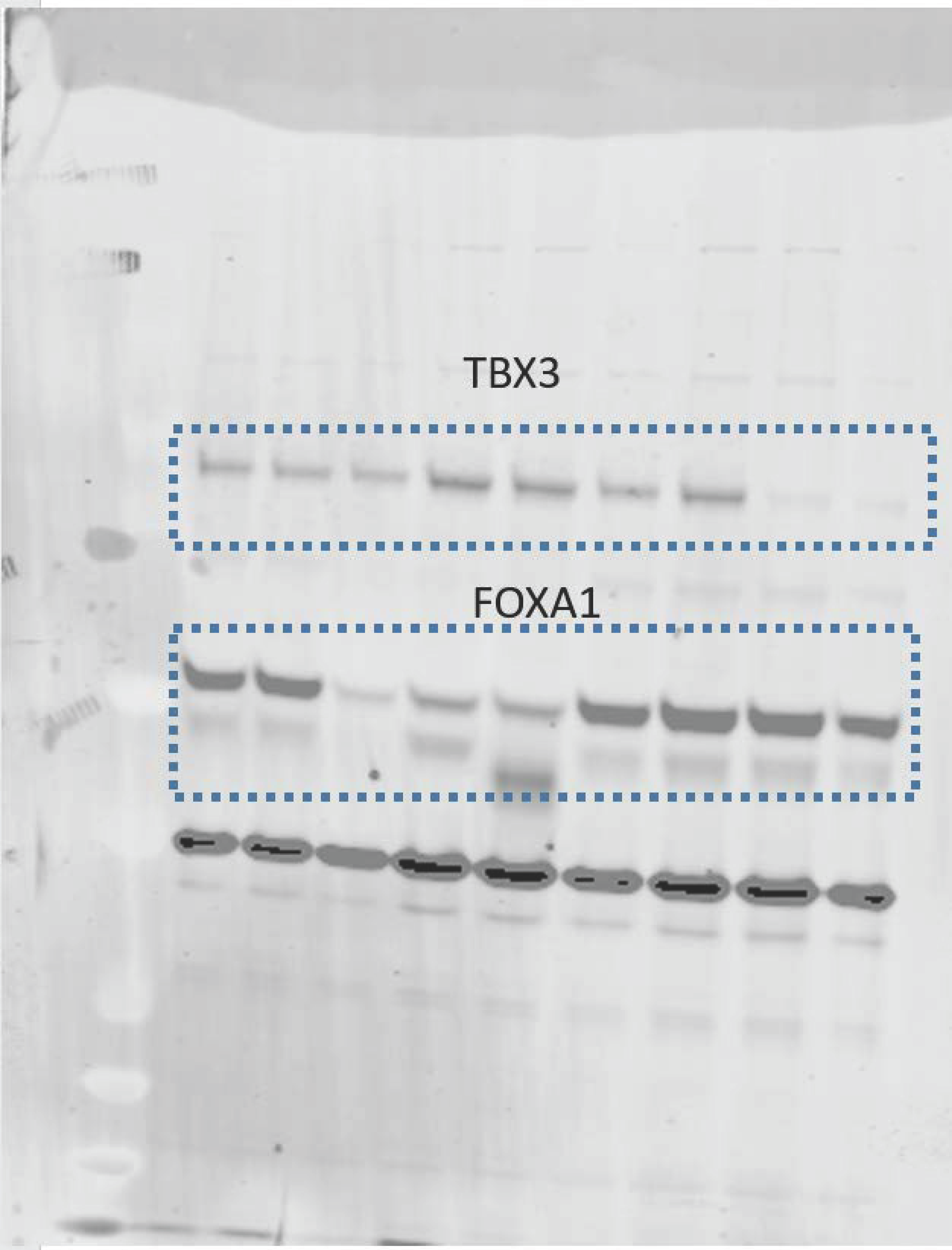

Supplement: Figure 4—source data 10. [file elife-73223-fig4-data10.zip › Figure 4 - Source Data 10.tif]

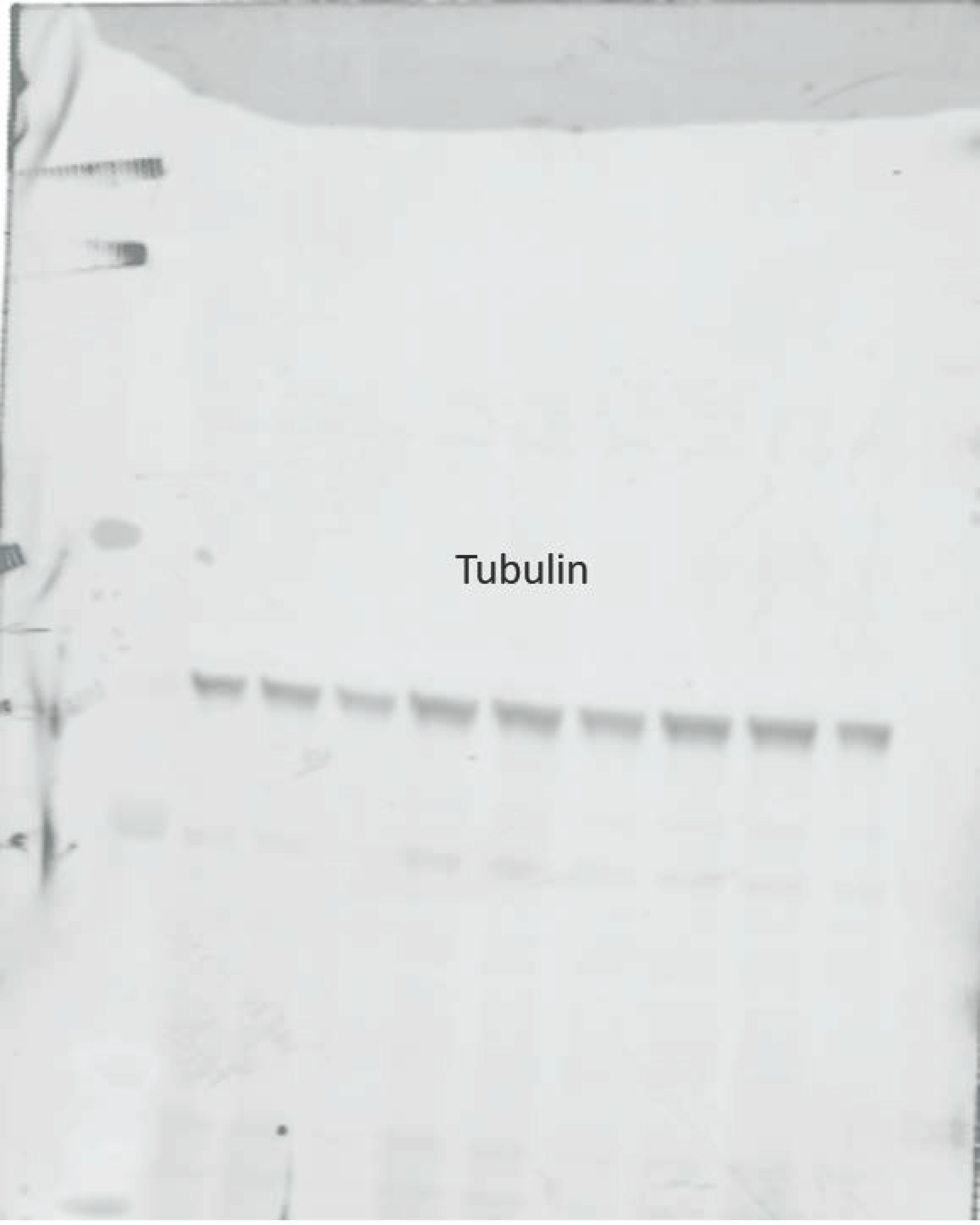

Supplement: Figure 4—source data 11. [file elife-73223-fig4-data11.zip › Figure 4 - Source Data 11.tif]

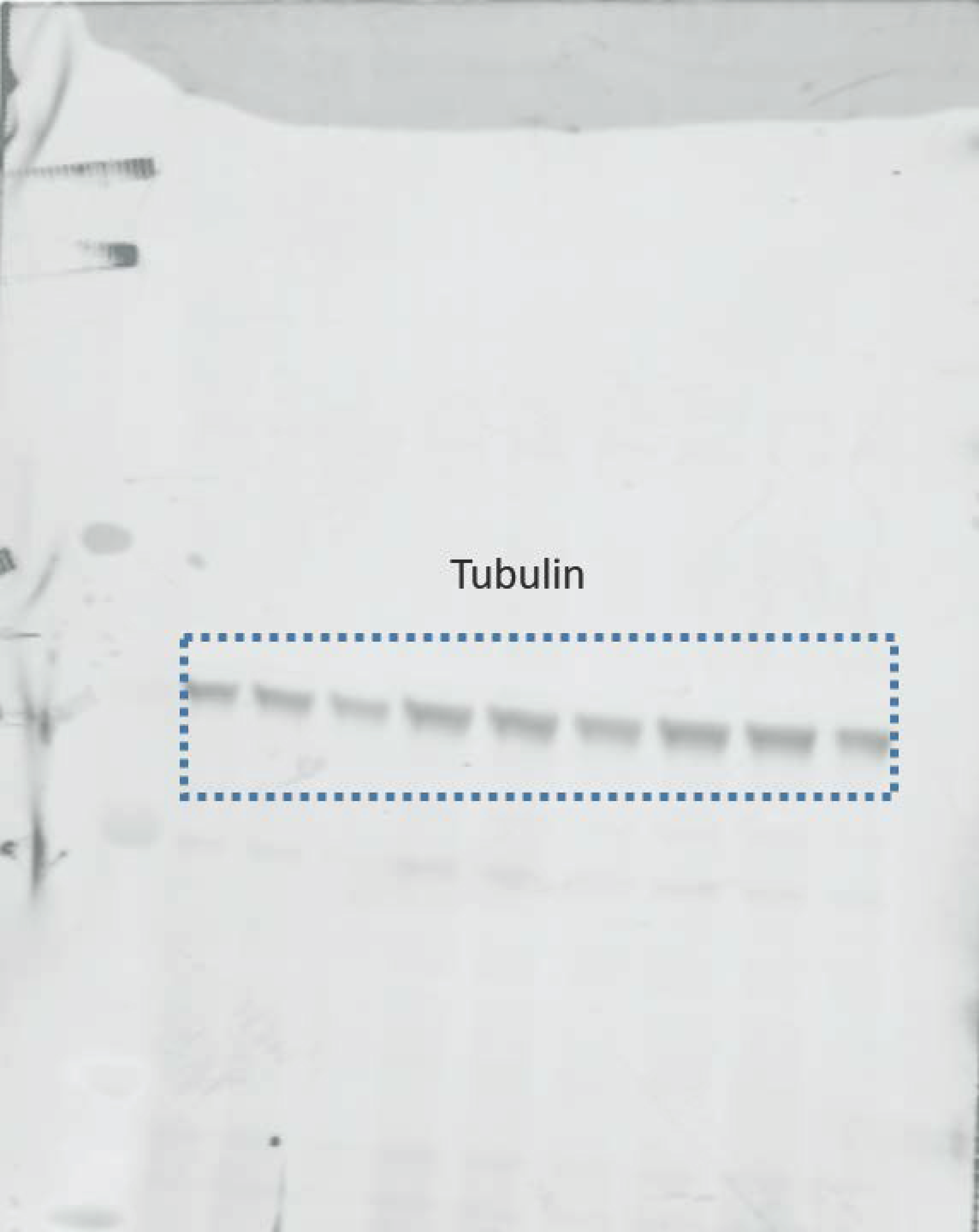

Supplement: Figure 4—source data 12. [file elife-73223-fig4-data12.zip › Figure 4 - Source Data 12.tif]

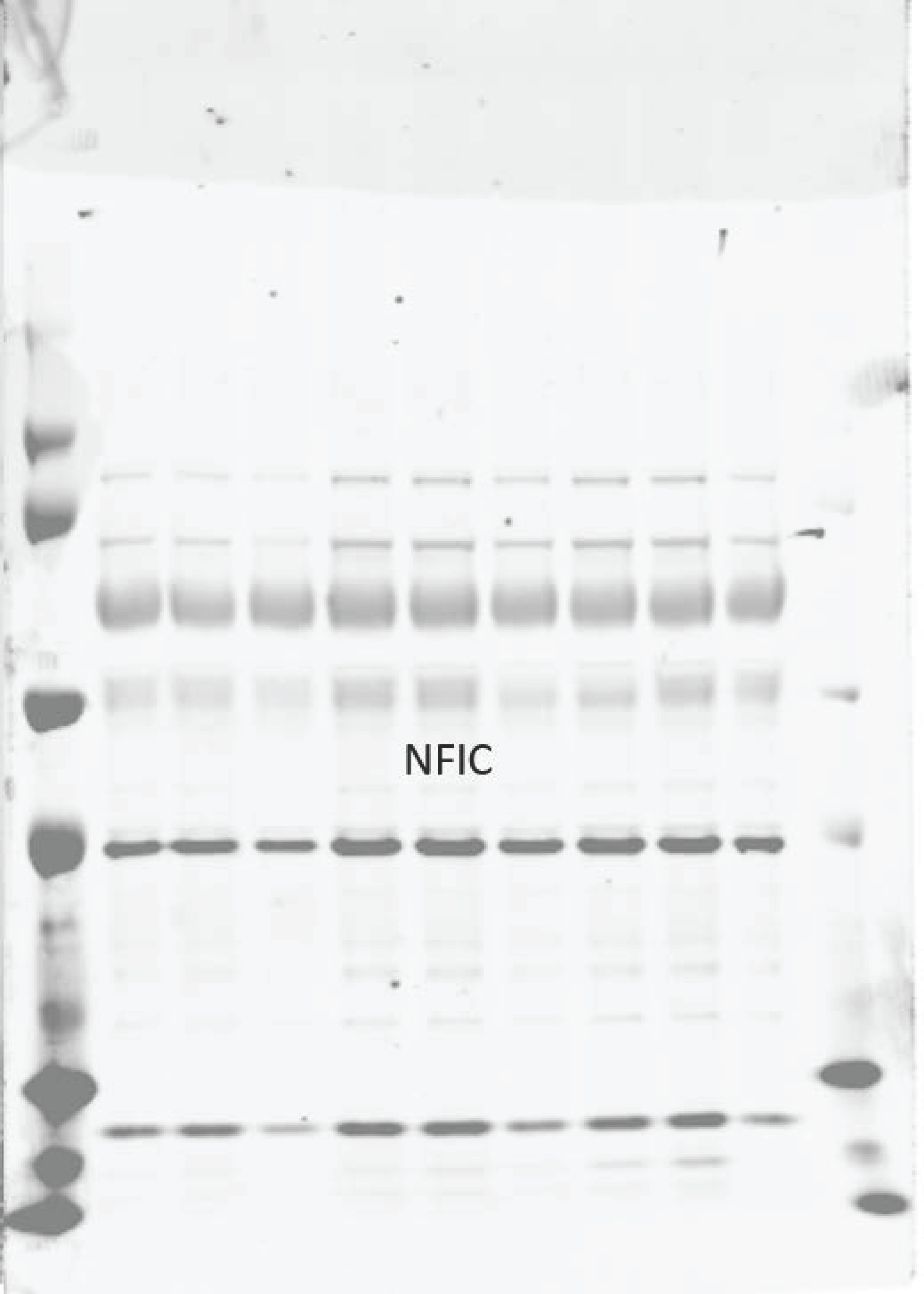

Supplement: Figure 4—source data 13. [file elife-73223-fig4-data13.zip › Figure 4 - Source Data 13.tif]

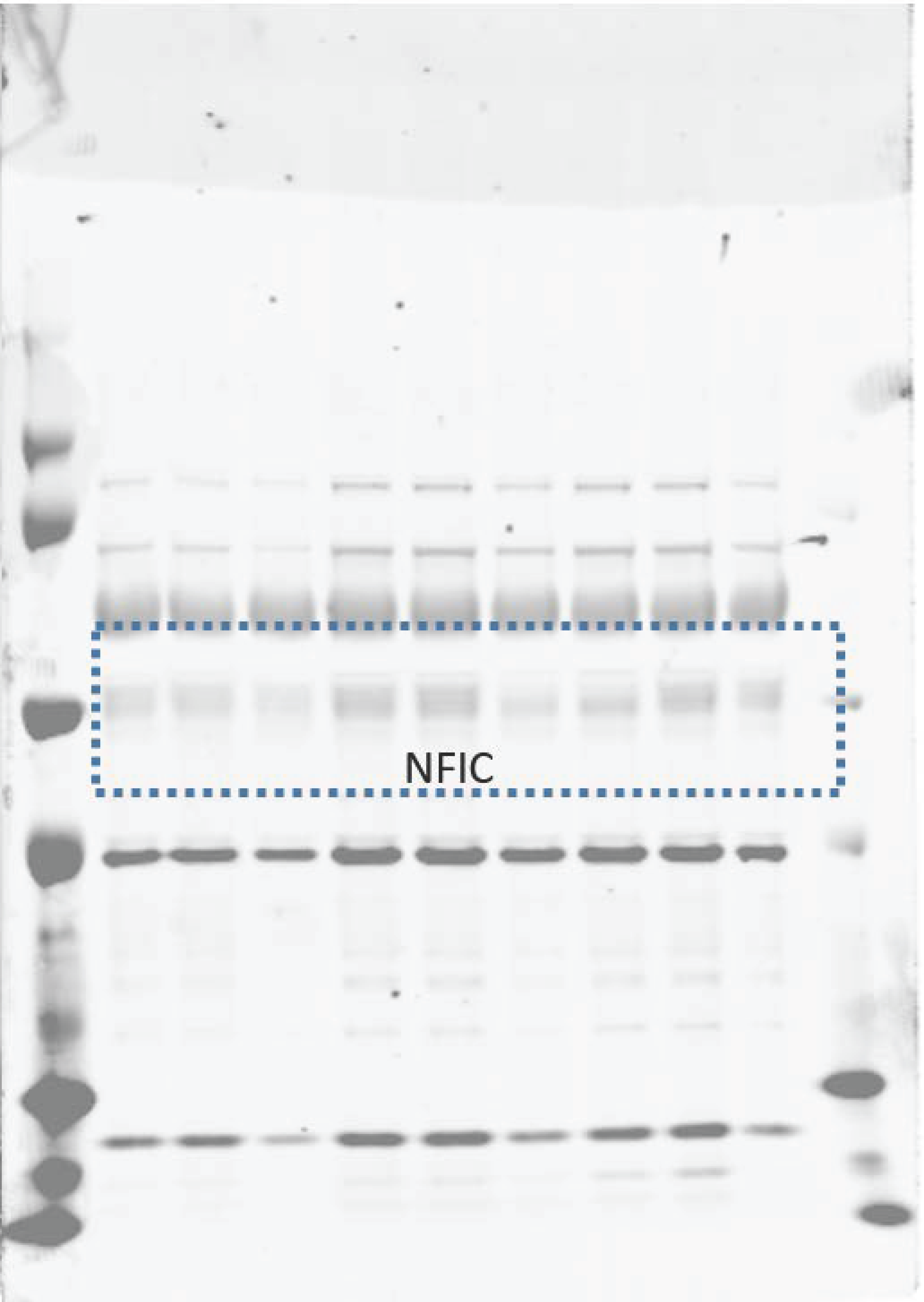

Supplement: Figure 4—source data 14. [file elife-73223-fig4-data14.zip › Figure 4 - Source Data 14.tif]
